# *PRO-Mueve Relaciones Sanas* – A Gender-Based Violence Prevention Program for Adolescents: Assessment of Its Efficacy in the First Year of Intervention

**DOI:** 10.3389/fpsyg.2021.744591

**Published:** 2022-01-03

**Authors:** Lilian Velasco, Helena Thomas-Currás, Yolanda Pastor-Ruiz, Aroa Arcos-Rodríguez

**Affiliations:** Department of Psychology, Universidad Rey Juan Carlos, Madrid, Spain

**Keywords:** prevention, gender-based violence, dating violence, adolescence, prevention programs

## Abstract

*PRO-Mueve Relaciones Sanas* (PRO-Mote Healthy Relationships) is a gender-based violence and dating violence prevention program targeted at adolescents. The program has been designed to be implemented during three consecutive courses [from the first to third year of Spanish mandatory secondary education (ESO)] in 8 annual sessions, imparted by university students who have been previously trained and supervised by university professors. The present study evaluates the effects of the program after the first year of implementation through a quasi-experimental design (Intervention Group *N* = 181; Quasi-control group *N* = 62; *M*_age_ = 12.11; SD_age_ = 0.57; 54.7% girls) and assesses whether there are gender differences in the outcomes. The results obtained evidenced a significant reduction in benevolent sexism in the intervention group compared to the quasi-control group. Regarding hostile sexism, it was found to increase significantly in the quasi-control group, while it remained stable in the intervention group. Thus, there were significant differences between both groups after the intervention. Likewise, romantic love myths were found to decrease significantly, and knowledge about gender-based violence increased significantly in the intervention group between the two time points assessed, although there were no significant differences with the quasi-control group. No gender differences in the outcomes of the program were observed. The obtained results supported the efficacy of the program during the first year of intervention in the first course of the ESO and laid the foundation for the following phases of intervention.

## Introduction

Gender-based violence (GBV) has significant negative consequences for those who suffer it, resulting in both physical and psychological damage, severe injuries and even death. Violence against women (VAW) has been normalized to some extent in society. This is a phenomenon that has been declared an epidemic by the World Health Organization (OMS, 2014a), since one in five women has suffered sexual abuse in childhood and one in three has been the victim of physical or sexual violence by a partner at some point in life (OMS, 2014b). In Spain, there was an 8.4% decrease in victims of GBV in 2020 (totaling 29.215 cases). Of these, 514 (−28.5%) were under 18 years of age [Instituto Nacional de Estadística (INE), 2021]. It is important to highlight that this decrease in the number of cases with respect to previous years is due to the situation of home confinement decreed by the state of alarm generated by the COVID-19. In the first months of 2021, the figures have shot up again: 24 deaths as of July 5, 2021 (Ministerio de Igualdad, 2021). Likewise, a macro survey conducted last year has shown that 21.5% of Spanish women have experienced physical violence over their lifetime and 13.7% have suffered some type of sexual violence (Delegación de Gobierno contra la Violencia de Género, 2020). Such data cause great social alarm and concern due to the effects and consequences for the future of women. In recent decades different studies have been made on the underlying causes, risk factors, and consequences - with the suggestion of preventive measures (Jennings et al., 2017; Gracia-Leiva et al., 2019). There is evidence that violence among adolescents and violence in dating behavior lies at the root of GBV in adult life (Wincentak et al., 2017).

Although there is no clear agreement on the definition of dating violence (DV), there is consensus that it refers to dating, i.e., relations between young people and between adolescents, and that there are generally three elements which are always present: (1) the phenomenon always occurs in the context of a dating relationship; (2) an attempt to control or dominate the partner is made; and (3) there is threat of or actual violence (Rubio-Garay et al., 2015).

There are several factors that appear to be associated with DV and GBV such as: gender stereotypes, that can be defined as widespread beliefs about the characteristics and attributes that women and men have or should have and the social functions that both should or must perform (Bosch-Fiol and Ferrer-Pérez, 2012; Ellemers, 2018); myths referred to romantic love, understood as the set of socially shared beliefs about the supposed “true nature” of love (Ferrer et al., 2010; Bisquert-Bover et al., 2019; Cerro and Vives, 2019); and sexism, conceived as a prejudicial attitude or discriminatory behavior based on the supposed inferiority or difference of women as a group (Cameron, 1977). Sexism is considered to have two aspects: hostile sexism, which includes the most visible forms of discrimination according to its definition, and benevolent sexism, in which there is a positive affective tone (toward women) and includes more subtle and even prosocial discriminatory behavior, such as protectionism. In both cases, the subordination of women to men is promoted (Lemus et al., 2008; Ramiro-Sánchez et al., 2018).

Other factors associated with DV are gender roles, understood as the part of the stereotypes prescribing what each gender should do and that condition the processes of socialization (Galán and Macías, 2019), structural inequality, power relationships and subordination (OPS, 2013), as well as abusive adult relationships (O’Keefe, 2005). Understanding these factors alongside the risk factors of DV is important in order to prevent such problems. Risk factors include interpersonal (biological, behavioral, psychological, and relational) and situational variables (family, social-community, physical, economic or historical environment) and may act as triggering, facilitating, modulating, protective or inhibiting factors (Vagi et al., 2013; Rubio-Garay et al., 2015).

On the other hand, the consequences of GBV manifest in four areas: (1) physical problems (World Health Organization, 2014; Pires et al., 2019), (2) mental health and behavioral problems (Ruiz-Pérez and Plazaola-Castaño, 2005; Ellsberg et al., 2008), (3) disorders related to sexual and reproductive health (Plazaola-Castaño and Ruíz-Pérez, 2004), and (4) a large group of chronic diseases (World Health Organization, 2014). Women who suffer from VAW have a worse health status than those who have not been victims of violence (Ruiz-Pérez et al., 2006; Sanz-Barbero et al., 2014). All these issues indicate that violence should be treated as a public health problem (Plazaola-Castaño and Ruíz-Pérez, 2004; Ruiz-Pérez et al., 2006; Vagi et al., 2013).

To prevent violence in relationships, preventive measures focused on changing attitudes and behaviors are the most appropriate strategy in order to mitigate and palliate its effects (Canet et al., 2019; Miller et al., 2020). The efficacy of DV prevention programs is increased when such programs are conducted in the educational setting - particularly in secondary education centers, where the first dating relationships develop (Rubio-Garay et al., 2017). Moreover, prevention programs that also focus on teacher and instructor training are more effective since they allow early intervention to reduce risks and protect those affected (Collins and Swearer, 2012).

The OMS (2014a) report compiled information on programs implemented in different countries with the purpose of preventing some of these forms of violence, including sexual violence, juvenile violence and dating violence. This report showed the advances in this field that have allowed for the implementation of preventive actions related to a specific gender violence situation. This established a starting point for preventive measures.

In Spain, legislation (Ley Orgánica 1/2004, 2004) on comprehensive protection measures against GBV establishes a series of preventive measures referred to this problem. In particular, title 1, chapter 1 of the educational setting, article 4, defines the principles and values of the education system. Based on this, different programs have been developed in different areas: school, family and community. This diversity ranges from short programs, talks or videos to other initiatives that also include programs covering several sessions with interactive exercises (De la Peña et al., 2011; Luzón et al., 2011; Fernández-González and Muñoz-Rivas, 2013; Mateos, 2013; Aroca Montolío et al., 2016; Delgado, 2017; Canet et al., 2019; Muñoz-Rivas et al., 2019a). These GBV prevention programs for adolescents in Spain have inspired some of the activities carried out by *PRO-Mueve*.

### PRO-Mueve Relaciones Sanas

#### Theoretical Background

PRO-Mueve Relaciones Sanas is a Spanish language primary prevention program that provides knowledge on gender-based violence and DV and promotes healthy relationships. From the perspective of the Social Learning Theory (Bandura, 1973, 1977), the antecedent and situational factors model of DV (Riggs and O’Leary, 1989) and the adoption of a deep gender perspective, we pay special attention to gender relations and their social reproduction as the basis of violence, as recommended by Santoro et al. (2018).

Preventive programs have proven to be the most effective resource in DV (Muñoz-Rivas et al., 2019b) and prevention strategies reduce, eliminate, and avoid the appearance of aggressive and violent behaviors in dating relationships (Foshee et al., 2005; Bell and Stanley, 2006; Wolfe et al., 2009). Implementing educational programs is one of the preventive measures that have shown efficacy in the prevention of DV (Jennings et al., 2017). Previous programs using multiple educational tools and different interactive activities (videos, role-playing, debates, or games) have obtained positive results reducing DV. These programs prioritize gender equality in intimate partner violence prevention (Foshee et al., 2005; Bell and Stanley, 2006; Díaz-Aguado, 2009; Garrido and Casas, 2009; Wolfe et al., 2009; De la Peña et al., 2011; Luzón et al., 2011; Fernández-González and Muñoz-Rivas, 2013; Mateos, 2013; Aroca Montolío et al., 2016; Delgado, 2017; Canet et al., 2019; Muñoz-Rivas et al., 2019b).

#### Objectives and General Characteristics

PRO-MUEVE was created as a structured program (step by step), with specific content for each session. It also covered the need to teach students in a practical way, as several authors have pointed out (Blázquez et al., 2009; De la Peña et al., 2011).

The program was designed to run for three consecutive academic years between the 1st and 3rd years of secondary education (12–14 years) and had three general objectives: (1) To learn to distinguish between a healthy relationship and an abusive and violent relationship; (2) To become aware of the link between factors associated with violence (gender stereotypes, myths of romantic love, sexism, gender roles, structural inequality, power, and subordination relationships, etc.), abusive relationships and their consequences (for both the victim and the perpetrator, and even their friends and family environment); and (3) To acquire skills to improve relationships (social skills, self-esteem; healthy ideation versus distorted cognition, self-regulation of emotions, anger management, adequate use of information technologies in relationships, adequate resolution of conflicts, etc.).

The results reported in this study therefore correspond to the first course in which the first objective and half of the second objective were covered. The specific objectives that were addressed in the first year of intervention were: (a) To learn that being treated with respect in a relationship is a legally recognized right, and that abuse is a criminal offense; (b) To know Human Rights, the Spanish Constitution and Act 1/2004 on comprehensive protection measures against GBV; (c) To distinguish situations of GBV according to the law, or other situations not present in the law – where there is a certain legal vacuum (sexual harassment, sexual violence, intra-gender violence, etc.), through every day and close examples; (d) To identify the characteristics and positively assess the benefits of a healthy relationship; (e) To raise awareness about gender inequalities and their relationship with GBV; (f) To recognize sexism as a factor associated to GBV; (g) To reduce sexist beliefs; (h) To identify and reduce myths and stereotypes regarding traditional gender roles, GBV and romantic love; (i) To learn to identify and reduce the different forms of micro-sexism; and (j) To know the resources available in the community for women care and victims of GBV in the adolescent population.

#### Description and Evaluation

PRO-Mueve sessions were delivered through a collaborative learning and active participation method based on practical and dynamic exercises, and fun activities such as games, role-playing, discussion groups, video clip watching and debates. In turn, to reinforce the knowledge acquired, at the end of each session the students received a poster with the contents covered in it. In addition, they were asked to reflect upon each session over the next 3 weeks in order to be prepared to ask questions they might have before the following session.

The purpose of this pilot study was to evaluate the effect of the first year of implementation on knowledge about gender-based violence and legislation, sexism attitudes, and myths of romantic love in adolescents. In addition, in response to the demand of some social organizations in our country that denounced that the programs were less effective for boys, our study also evaluates whether the program exerts a differential effect upon the girls and boys enrolled in it. This research question was prompted by the comments of some professionals and researchers in the field indicating that boys tend to be less involved in programs of this kind, and therefore benefit comparatively less than girls (Exner-Cortens et al., 2019). However, there is precedent indicating that prevention-oriented programs in the context of secondary education have not found gender differences in their effectiveness (Taylor et al., 2010).

### Hypotheses

Taking previous studies of prevention programs in this population as a starting point (Hernando, 2007; Garrido and Casas, 2009; Muñoz-Rivas et al., 2010; Díaz-Aguado and Carvajal, 2011; Mateos, 2013; Muñoz et al., 2013), and previously identifying the factors that appeared associated with GBV, we hypothesized that students who complete the program during the first year of intervention will significantly reduce their sexist beliefs, myths of romantic love, and will increase knowledge on gender-based violence and legislation compared to the quasi-control group.

## Materials and Methods

### Participants

**FIGURE 1:**
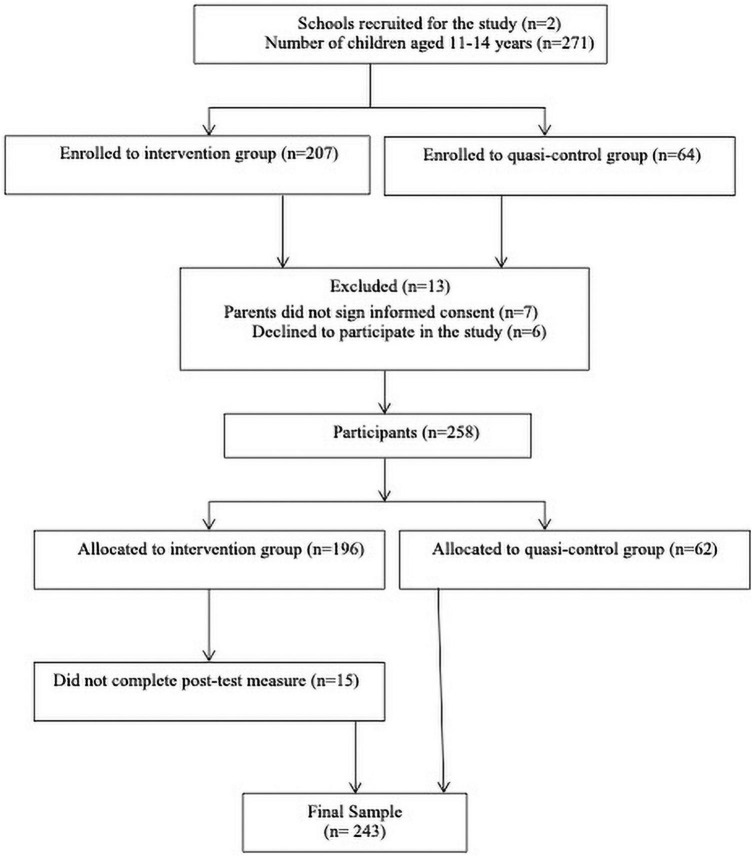
Participants’ flow diagram.

Two-hundred and seventy-one first course of High School students were eligible to participate in this study, with 207 participants belonging to the intervention group and 64 to the quasi-control group. Of these, 15 participants did not complete measures at the end of the intervention, mainly because they were not in class on the day of data collection. Six adolescents declined to participate in the study, and seven parents did not provide consent (see Figure 1). Thus, 243 participants were allocated. One hundred and eighty-one belonged to the intervention group and completed the program and assessment measures (pre-test and post-test) and 62 belonged to the quasi-control group and filled out the two assessment measures. The students aged from 11 to 14 years (*M* = 12.11, SD = 0.57) and 133 were girls (54.7%) and 110 were boys (45.3%).

Previously, a power analysis had been used to determine the minimum sample size required, using the program G*Power 3.1.9.7. For a repeated measures ANOVA design (within-between interaction) with a medium effect size (f squared = 0.15), two recognized groups (intervention vs. quasi-control group), four repeated measurements (pre-test and 3 post-test), with a significance level of 0.05 and a power of 0.95, an overall sample size of 98 subjects was obtained. Respecting these conditions, with a composition of 49 persons in each group (intervention and quasi-control), the design was sufficiently well supported. Unfortunately, the pandemic and the confinement prevented us from carrying out all the post evaluations we had planned (at the end of the program, at 6 months and at 1 year). In addition, comparisons between conditions showed that there were no differences by sex (χ^2^ = 0.373, df = 1, *p* = 0.541) or age (*U* = 5059.50, *p* = 0.101) between the intervention and quasi-control group, which makes the two groups comparable at least in these characteristics.

### Study Design and Procedure

We used a quasi-experimental pre-post design with a quasi-control group. The sample was non-probabilistic, and participation was voluntary. Participants who belonged to the intervention group completed the *PRO-Mueve* program. It was delivered in nine sessions, one every 2–3 weeks, from November 2018 until June 2019. The pre-test evaluation was the first session, and the post-test evaluation was the last session. The program was carried out in 7 1-h sessions, using active learning methodologies. Both interventions and assessment measures were administered by expert professors and research assistants.

Recruitment was carried out in the city of Pinto (Madrid) at the end of the 2017–2018 academic year. Pinto is a town of just over 42,000 inhabitants located in the south of the Community of Madrid. After one meeting with the councilors of all schools of the locality (3), two of them were interested in applying the program in the 2018/2019 academic year through the entire course. Eligible participants were those who belonged to the first course of high school (*N* = 271). A quasi-randomization was performed. Three classes were randomly assigned to the quasi-control condition (*N* = 64) and nine (*N* = 207) to the intervention condition. The groups remained intact with their corresponding class during the intervention or assessment sessions. The quasi-control group did not receive any intervention and it is smaller because both schools wanted their students to participate in the program.

The program sessions for the intervention group were run during normal class time by two psychologists who had leading roles and a research assistant. Leaders were supervised by expert professors from the University and for each session they conducted a 1-day accredited training course with the research assistant to explain the content before each session.

### Survey Administration and Human Subject Protections

With the approval of the researchers’ university and in accordance with school policies, parents provided passive consent and students gave active assent to complete questionnaires. The schools notified parents that their child’s school would be taking part in a research study, providing information and a contact number for any questions, concerns, or to exclude their child from the survey data collection. Students were also informed that the questionnaires were part of a university research project, their participation was voluntary, and answers would remain confidential. Students signed an assent to participate. The research team received seven reports of parents denying consent, and six students declined to provide assent to the survey data collection. They were assigned to a classroom for studying during the program implementation sessions.

Before delivering the program, students from both groups (intervention and quasi-control) completed a 1-h self-administered pre-test questionnaire. Seven months later, after the intervention group completed the program *PRO-Mueve Relaciones Sanas*, all students completed a post-test questionnaire. Because the program implementation and survey data collection were conducted as part of regular school activities, nearly all enrolled students were present at both the pre-test and post-test. The research assistants conducted data collection and data entry and sent the university researchers a data set without any individually identifying information. We matched student pre-tests to post-tests with a unique confidential identifier that students created for themselves, using combinations of numbers and letters.

The study followed (Ley 14/2007, 2007) the Spanish Biomedical Research Act (2007) and was approved by the Clinical Research Ethics Committee from the King Juan Carlos University (code: 0404201 806318).

### Curriculum Training and Delivery

Implementation was conducted by two leaders in each group. Every session included a research assistant. All leaders attended a 1-day accredited training course every 3 weeks before the sessions to explain the session content to the research assistants. Training was led by expert professors from the University. There were eight group leaders in total. All the group leaders were psychologists who were enrolled in the master’s degree in General Health Psychology or who were in the last course of the degree in Psychology or Criminology, or in the dual degree in Psychology and Criminology. It was decided that the program would be conducted by young people in order to ensure a lesser intergenerational distance between the instructors and adolescents, and thus facilitate greater attention and identification with the instructors. As indicated by social learning theory, adolescents learn better when they are among each other. As evidenced by drug prevention or similar programs, when the goal is to maintain desired behaviors, young people are more potent models (Berger, 2015; Aroca Montolío et al., 2016) imitating those perceived to be more similar (Edinyang, 2016).

The professors responsible for the program, experts in social psychology and clinical psychology, organized a DV training course (3 sessions of 4 h each) at the university in which more than 50 students participated on a voluntary basis and obtained credits. From this course onward, we asked for volunteers who wanted to participate in the implementation of the program, in exchange they were given a diploma certifying their participation and some of these students could even do their master’s degree thesis on it. With this small group of volunteers, a 2-h training session was held prior to each session with the program materials and discussions were held on the implementation of each session. Both training and implementation had no cost for the schools. The material (photocopies and stationery) was provided by the city council.

### Outcome Measures

A set of measures was selected from previous studies with an adolescent population. As this is a primary prevention program that starts before adolescents enter their first relationships, the selected indicators of change refer to attitudes and knowledge.

The *Gender-based Violence Questionnaire* designed by Mateos (2013) was administered to assess knowledge about violence in interpersonal relations, gender-based violence and legal aspects related to violence. Therefore, this instrument was used to assess knowledge about GBV. This questionnaire consists of 12 multiple-choice questions with three possible answers, of which only one alternative is correct. An example of these items is: “When I hear about gender-based violence, I think it is: (a) Violence directed by men toward women due to the fact of being a woman, (b) Violence that occurs only within the home, and (c) Violence between men and women.” The score obtained by each subject reflects the number of correct answers given.

*The myths of romantic love scale* developed by Ferrer Pérez et al. (2010) was used to assess myths of romantic love. This instrument consists of 10 items that assess 7 myths based on their description or some established clichés about them. Specifically, the myths assessed are: Myth of the better half (item 1), Myth of eternal passion (item 2), Myth of omnipotence (items 3 and 7), Myth of marriage (item 4), Myth of pairing (items 5 and 6), Myth of jealousy (item 8) and Myth of ambivalence (items 9 and 10). For each item, the degree of agreement is measured by means of a 5-point Likert scale from 1 (*Complete disagreement)* to 5 (*Complete agreement)*, where the higher the score, the greater the degree of acceptance of the myth. The overall average score on the scale was used. The internal consistency presented for this study showed a Cronbach’s alpha coefficient of 0.57 for pre-assessment and 0.66 for post-assessment.

To assess sexism, we administered the Spanish adaptation (Lemus et al., 2008) of the *Ambivalent Sexism Inventory* (ASI; Glick and Fiske, 1996). This instrument measures the presence of both hostile and benevolent sexist attitudes. It consists of 20 direct items (10 referred to hostile sexism and 10 to benevolent sexism) measuring the degree of agreement with different sexist statements based on a 6-point Likert scale from 1 (*Strongly disagree*) to 6 (*Strongly agree)*. The overall average score on each subscale was used. The psychometric properties were found to be adequate. In this study, the internal consistency indices ranged from 0.81 to 0.86.

### Analyses

The levels of missing data ranged from 0 to 5% in the different items of the scales. The SPSS multiple missing value imputation procedure was used. Twenty different imputations of the missing data were calculated as recommended by Graham et al. (2007). An average score of the different imputations was then calculated for each of the variables.

Preliminary analyses were carried out. These consisted of comparing whether there were significant differences in the assessed variables in the students of the two centers (T1) and calculating the descriptive statistics of the variables of the intervention group and quasi-control group at the two assessment time points (T1, T2). We also calculated correlations between the same assessed variables at the two time points (T1, T2).

To evaluate the effectiveness of the program, a multivariate analysis of variance (MANOVA) was carried out with these data. Three independent variables were used: time (pre-post measures); condition (intervention and quasi-control groups) and gender (males and females). We were interested in assessing the possible interaction between the gender program and the condition group. The dependent variables considered were basic knowledge about gender-based violence, sexism attitudes, and myths of romantic love.

To study the effect of the independent variables we selected Pillai’s criteria, as this is considered the most robust measure against violations of statistical assumptions (Tabachnick and Fidell, 2007). Partial eta squared (η^2^) was used to estimated effect size in accordance with Cohen (1992), who considered a value close to 0.02, 0.13, or 0.26 as indicators of a small, medium or large effect sizes, respectively. The assumption of sphericity of the data was assessed, in all variables ε = 1 was obtained, so no correction was applied to the *F* value. In order to clarify the significance of the time × condition interaction effect, pairwise comparison tables and the significance level of the differences with Bonferroni adjustment were requested. For all the analyses, the statistical program version 26 of SPSS was used.

## Results

### Preliminary Analysis

Regarding the differences between the collaborating centers in the first evaluation (T1), the *T*-tests indicated that there were no significant differences between the centers in hostile sexism, *t*(241) = 1.7, *p* = 0.076, *d* = 0.16; benevolent sexism, *t*(241) = −1.2, *p* = 0.203, *d* = 0.22; romantic love myths *t*(241) = 0.1, *p* = 0.844, *d* = 0.02 or in the knowledge about gender-based violence, *t*(241) = 0.7, *p* = 0.456, *d* = 0.08. In all cases, the Cohen’s effect size was small (values between 0 and 0.20) or adopted a value close to this range (Cohen, 1988).

The descriptive statistics for the different variables in both conditions (quasi-control and intervention group) according to the two temporary measures (T1 = pre-test; T2 = −post-test) are summarize in Table 1.

**TABLE 1:** Univariate statistics for variables in analysis.

	Quasi-control group *N* = 62		Intervention group *N* = 181	
Variables	T1 Mean (SD)	T2 Mean (SD)	T1 Mean (SD)	T2 Mean (SD)
Hostile sexism	2.24 (0.81)	2.48 (1.11)	2.10 (0.81)	2.02 (0.89)
Benevolent sexism	2.77 (0.99)	2.72 (1.03)	2.78 (1.03)	2.19 (1.01)
Myths of romantic love	2.60 (0.45)	2.58 (0.55)	2.65 (0.47)	2.42 (0.55)
Knowledge about GBV	8.01 (1.50)	7.95 (1.91)	7.74 (1.41)	8.36 (1.51)

Moreover, a simple bivariate correlation was computed between T1 and T2 in all the measures. Table 2 shows that the correlations adopted lower values for the intervention group in almost all the measures considered. Contrary to what is expected, a curious fact was the greater correlation observed in the hostile sexism variable between T1 and T2 in the intervention group.

**TABLE 2:** Pearson bivariate correlation of measures (T1, T2).

Variables	Quasi-control group	Intervention group
Hostile sexism	0.443**	0.595**
Benevolent sexism	0.664**	0.491**
Myths of romantic love	0.485**	0.351**
Knowledge about GBV	0.495**	0.315**

**p < 0.001.

### Effects of the Intervention

A MANOVA of repeated measures analysis showed a significant effect of time × condition interaction, Pillai’s trace = 0.098, associated with *F*(6,236) = 6.422, *p* < 0.001, η^2^ = 0.098; while time × condition × sex interaction was not statistically significant, Pillai’s trace = 0.027, associated with *F*(4,236) = 1.662, *p* = 0.161, η^2^ = 0.027.

Considering univariate effects (of time × condition interaction), significant differences were found in the following variables: hostile sexism, *F*(1,239) = 8.700, *p* = 0.003, η^2^ = 0.035; benevolent sexism, *F*(1,239) = 14.476, *p* < 0.001, η^2^ = 0.057; myths of romantic love, *F*(1,239) = 4.642, *p* = 0.032, η^2^ = 0.019; and knowledge about gender-based violence, *F*(1,239) = 11.363, *p* = 0.001, η^2^ = 0.045.

Table 3 shows the pairwise comparisons of the levels of each variable evaluated in both conditions (quasi-control versus intervention) within the time factor. It can be observed that there were no significant differences in the pretest (time 1) between the intervention group and the quasi-control group in the different variables evaluated (hostile sexism, benevolent sexism, myths, and knowledge about GBV). On the other hand, significant differences were found between both groups after the intervention phase (time 2) in the hostile sexism and benevolent sexism variables. In the case of myths and knowledge about gender-based violence, the differences between the two groups (time 2) were not statistically significant.

**TABLE 3:** Paired comparisons of means time × condition.

Measure	Time	(I) Condition	(J) Condition	Mean difference (I-J)	Error Desv.	Sig.^b^	95% confidence interval for difference^b^	
							Lower limit	Upper limit
Hostile Sexism	1	Quasi-control	Intervention	0.143	0.118	0.226	–0.089	0.376
	2	Quasi-control	Intervention	0.511*	0.135	0.000	0.246	0.777
Benevolent sexism	1	Quasi-control	Intervention	–0.023	0.149	0.875	–0.317	0.270
	2	Quasi-control	Intervention	0.530*	0.150	0.001	0.234	0.827
Myths of romantic love	1	Quasi-control	Intervention	–0.045	0.070	0.523	–0.182	0.093
	2	Quasi-control	Intervention	0.135	0.079	0.090	–0.021	0.291
Knowledge about GBV	1	Quasi-control	Intervention	0.289	0.215	0.179	–0.133	0.712
	2	Quasi-control	Intervention	–0.433	0.241	0.073	–0.907	0.040

*Based on estimated marginal means.*

*The difference in means is significant 0.05.

*^b^Adjustment for various comparisons: Bonferroni.*

Table 4 shows the pairwise comparisons of the levels of each assessed variable at both time points (T1 versus T2) within the condition factor (intervention or quasi-control group). No significant differences were observed in the intervention group in hostile sexism between T1 and T2, but they were observed in the quasi-control group, showing that SH increased between the two time points. On the other hand, in benevolent sexism, significant differences were observed in the intervention group between time 1 and 2, indicating a decrease after the program sessions. Likewise, in the case of romantic love myths, a decrease in these myths was observed in the intervention group after the program (T2) and also an increase in knowledge about gender-based violence in adolescents in the intervention group after the program sessions (T2).

**TABLE 4:** Paired comparisons of means condition × time.

Mean	Condition	(I) Time	(J) Time	Mean difference (I-J)	Error Desv.	Sig.^b^	95% confidence interval for difference^b^	
							Lower limit	Upper Limit
Hostile sexism	Intervention	1	2	0.081	0.063	0.198	–0.042	0.204
	Quasi-control	1	2	−0.287*	0.108	0.008	–0.500	–0.075
Benevolent sexism	Intervention	1	2	0.596*	0.073	0.000	0.452	0.739
	Quasi-control	1	2	0.042	0.126	0.741	–0.206	0.290
Myths of romantic love	Intervention	1	2	0.225*	0.042	0.000	0.143	0.307
	Quasi-control	1	2	0.046	0.072	0.528	–0.097	0.188
Knowledge about GBV	Intervention	1	2	−0.608*	0.128	0.000	–0.861	–0.356
	Quasi-control	1	2	0.114	0.222	0.606	–0.322	0.551

*Based on estimated marginal means.*

*The difference in means is significant 0.05.

*^b^Adjustment for various comparisons: Bonferroni.*

**FIGURE 2:**
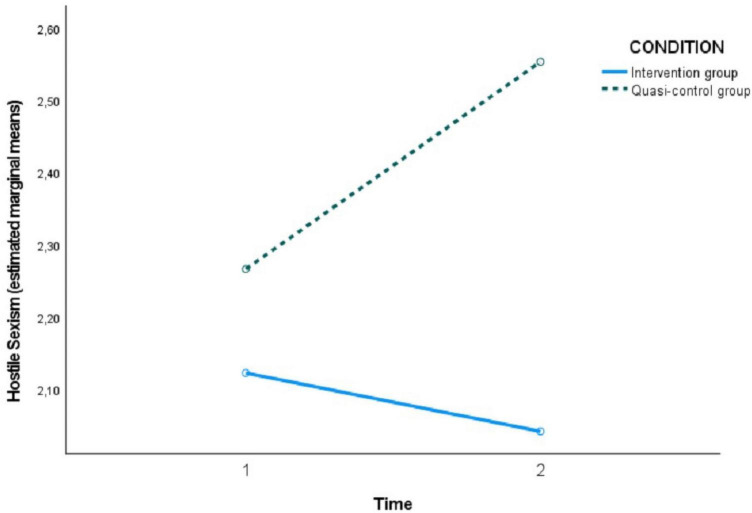
Effect of time × condition interaction on hostile sexism.

**FIGURE 3:**
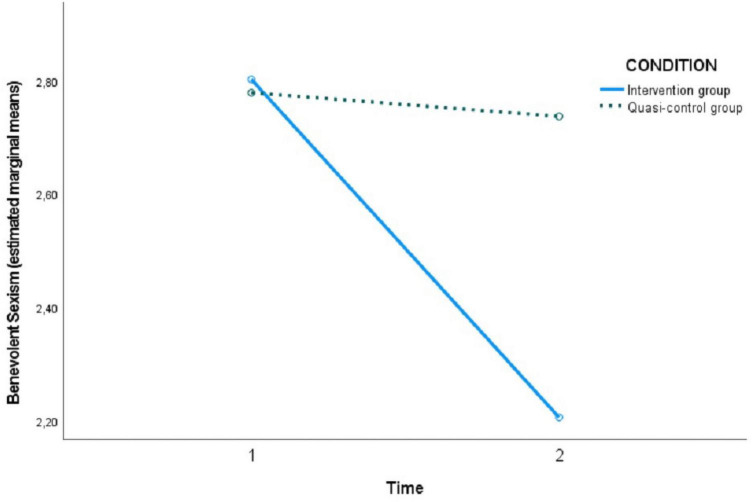
Effect of time × condition interaction on benevolent sexism.

**FIGURE 4:**
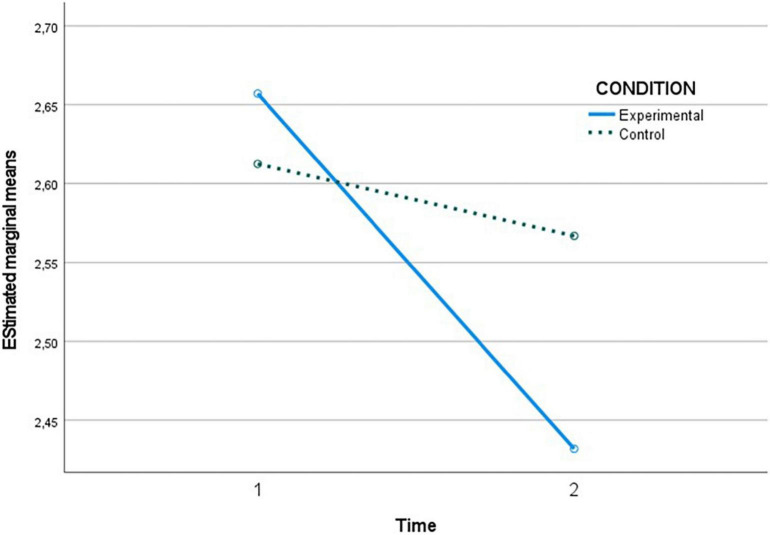
Effect of time × condition interaction on myths of romantic love.

**FIGURE 5:**
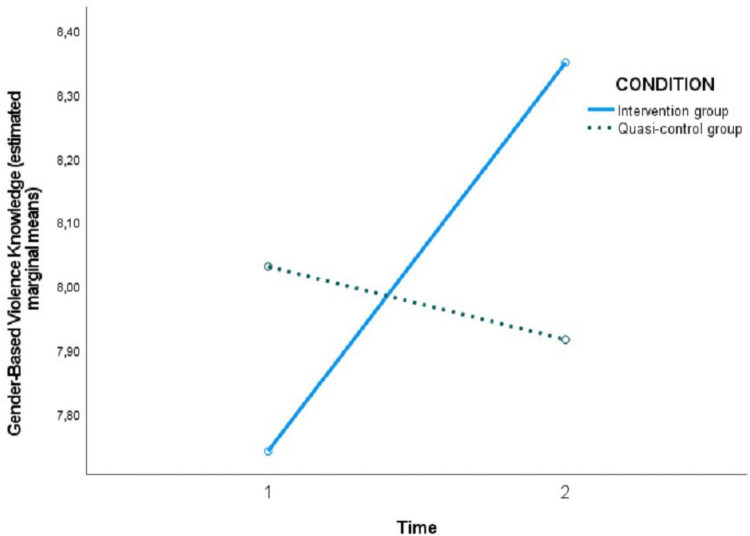
Effect of time × condition interaction on gender-based violence knowledge.

In the following Figures 2–5, the effect of the time × condition interaction described above can be visualized for each of the variables evaluated. The figures show an increase in hostile sexism in the quasi-control group between T1 and T2 and a slight non-significant decrease in the intervention group (Figure 2), a significant decrease in benevolent sexism (Figure 3) and romantic love myths (Figure 4) and an increase in knowledge about gender-based violence (Figure 5) in the intervention group after the program sessions (T2).

## Discussion

*PRO-Mueve* is a gender-based violence and dating violence prevention program targeted at adolescents. The program differs from others (e.g., Díaz-Aguado, 2009; Mateos, 2013) in that it comprises 7 annual intervention sessions and two sessions for evaluation implemented over three consecutive courses from the first to the third year of Spanish Mandatory Secondary Education. The present study reports the evaluation of the first year of intervention, in which the first objective and part of the second objective were developed. The program was developed in the school setting of the adolescents by university students in their final year who had received specific training to impart each session. Other experiences such as that of Aroca Montolío et al. (2016) underscore the importance of using references of similar age or peers for better assimilation of the prevention programs.

Our results evidenced changes in the intervention group between pre and post-intervention, showing a significant decrease in the beliefs of myths of romantic love and an increase in general knowledge about GBV and current legislation. We also found changes in the intervention group in comparison with the quasi-control group in benevolent sexism. These changes supported our hypothesis. However, with respect to hostile sexism our data reported an increase in it in the quasi-control group between the two time points, while it remained almost stable in the intervention group. Thus, significant differences appeared between the two groups after the intervention period. Few studies have been conducted on how sexist beliefs change during the adolescent stage. Blázquez-Alonso et al. (2016) reported an influence of the cognitive-social strategies developed at this stage, so that those adolescents with more prosocial strategies tended to reduce sexism to a greater extent (Blázquez-Alonso et al., 2021), whereas those with antisocial or asocial cognitive strategies firmly maintained or even exacerbated their sexist attitudes, these being of a more hostile nature. Other studies have shown a tendency or inertia to maintain hostile attitudes (Ferragut et al., 2016; Blázquez-Alonso et al., 2021). In addition, Blázquez-Alonso et al. (2016) reported an influence of the cognitive-social strategies developed at this stage, so that those adolescents with more prosocial strategies tended to reduce sexism to a greater extent (Blázquez-Alonso et al., 2021), whereas those with antisocial or asocial cognitive strategies firmly maintained or even exacerbated their sexist attitudes, these being of a more hostile nature This could explain why our data did not show a significant reduction in hostile sexism in the intervention group and it even increased in the quasi-control group. In line with previous studies (Bell and Stanley, 2006; Díaz-Aguado, 2009; Murta et al., 2013), our results also showed a decrease in benevolent sexism beliefs, although it is a variable that is persistent with age (Hammond et al., 2017). While sexist beliefs reinforce aggressive and violent behaviors, promoting more egalitarian beliefs and gender roles would favor healthy relationships based on good behavior.

Prevention is the best strategy for mitigating sexism, patriarchal domination and GBV, and for preventing the continuation of an unequal and unfair society (Canet et al., 2019). Furthermore, it should not be forgotten that one of the main reasons for failing to report GBV and for underestimating the severity of aggression is the existence of cultural factors focused on normalizing violence and maintaining a sexist culture in the population (Monreal-Gimeno et al., 2014; Pérez, 2015). To change this trend, more educational intervention is needed in schools from an early age, aimed at gender coeducation. Interestingly, the data from our study also showed a score on sexism, both hostile and benevolent, below the mean of this instrument, both in the pre-test and post-test (e.g., Lemus et al., 2008). This could be explained by the fact that this was an incidental sample that was not representative of the local adolescent population. It could also be explained by the presence in this particular municipality of gender agents who have been raising awareness in this regard for years or even by a change of trend in our context given that previous studies date back a decade. Further studies with representative samples would be necessary to reach a clear conclusion.

Also, the myths of romantic love, especially those linking love with control and jealousy, underpin violence in romantic relationships (Cava et al., 2020). For this reason, most GBV prevention programs in adolescence are aimed at reducing them. Our data support a significant reduction in these beliefs in the intervention group as a result of the program sessions. Both myths of romantic love and sexism are antecedents of GBV (Illescas et al., 2018). These beliefs sustain or precipitate violent behavior that would be unacceptable under normal circumstances (Caro and Monreal, 2017). Myths in turn contribute to accentuate sexism and perpetuate gender roles characterized by dominance-submission. This association is considered to be relevant, particularly in adolescence, because it may have a negative impact upon adolescents, generating emotional problems, differences in self-esteem construction, risk behaviors, unequal and dominance-submission based dating relationships and - in the most extreme situations - it may lead to physical, psychological and sexual violence (Bisquert-Bover et al., 2019). In this regard, our data support the results of previous programs in adolescents, such as those of Hernando (2007), Garrido and Casas (2009), and Muñoz-Rivas et al. (2010), in which romantic myths were reduced or eliminated.

It should be noted that the program described in the present study resulted in an increased understanding of GBV in the intervention group, with a distinction of the different types of violence and their legislation, as well as an awareness of the legal consequences of such behavior (general knowledge about gender-based violence). These data are in line with those obtained in previous programs, (Hernando, 2007; Garrido and Casas, 2009; Muñoz-Rivas et al., 2010; Fernández-González and Muñoz-Rivas, 2013; Mateos, 2013) reporting an increase in knowledge on gender violence, in attitudes of rejection of violence, and in the ability of adolescents to distinguish between a healthy relationship and an abusive one. In this program, we aimed to bring the legislative framework closer to adolescents, since few programs address this aspect (Mateos, 2013). Furthermore, providing this information in an entertaining, interactive, and dynamic way would fit the needs of the adolescent population. Although an increase in knowledge does not necessarily lead to a reduction in violence, we consider it a necessary starting point to continue with the next phases of the intervention, as it is a first step to raise awareness about the problem (following the I change-model, De Vries, 2017).

Based on Cohen’s criteria (1992), the program as a whole can be considered to exhibit a low effect size. It should be noted that if we look at the overall effect size of the program (intrasubject effect of the condition) this reaches a value of 9.8%. It should also be considered that this is the effect of the first year of the intervention; the full program will require assessing the effect of the intervention in all three years.

In addition to the efficacy of the intervention, we aimed to determine whether the program showed differences in its efficacy according to gender. Our data evidenced no significant differences in this respect, however, the program has shown to have similar effects among boys and girls, on the assessed variables, in line with a previous study of similar characteristics (Taylor et al., 2010). In contrast to these findings, some previous programs such as those developed by Garrido and Casas (2009), Aroca Montolío et al. (2016), and Muñoz-Rivas et al. (2019b) have reported greater benefit among girls than in boys. Some authors explain these differences by giving greater awareness of gender-based violence in the case of girls during adolescence (Garrido and Casas, 2009). *PRO-Mueve* intends to be a program that implicates boys and girls equally in such a significant social issue and seeks to make boys see that they can also become victims or witness abuse in their close circles. Our findings indicate that both girls and boys benefit from the program, contrast with those of other studies which state that girls benefit more than boys (Aroca Montolío et al., 2016).

This objective of making the program effective for both boys and girls, is one of the main goals of diversity. The program was applied in class groups from two public high schools, reaching all academic and social levels of the center, including immigrants and students with learning difficulties. It should be noted that we have not assessed the differential effectiveness of the program with these groups, this being a future objective to pursue with this program. Likewise, taking the program to a variety of centers with diverse socio-demographic characteristics in the Region of Madrid is another of our future goals. The achieved results are the first step in guaranteeing the success of the program’s dissemination.

The present study has some limitations that should be borne in mind. Firstly, it was carried out based on an incidental sample. Because of this, and due to the requirements of the centers that wished to collaborate, the size of the quasi-control group was smaller than that of the treatment group (30%). Secondly, mention must be made of the low reliability obtained in the questionnaire referred to myths of romantic love. In successive evaluations, additional measures should be used apart from self-reporting in order to allow more complete evaluation in this area. Thirdly, we did not assess whether the participants were in a relationship or have suffered from DV. Based on Hunter (2009), the pattern of violence in relationships occurs after the age of 17 and our sample ranges from 11 to 14. However, in the subsequent implementation of the program, we will evaluate these aspects. Fourthly, there is a lack of follow-up data to ascertain whether the benefits of the intervention are sustained over time. Fifthly, the changes generated by the program are not broad enough, and more sessions are required to consolidate them and produce greater changes in the variables evaluated. Finally, the implementation of such programs should also be made from the body of teachers and parents, thereby reinforcing the preventive measures (Canet et al., 2019). It is also necessary to implement the program in other samples in order to test the replicability of the present findings.

One of the main strengths of this study is the versatility of the intervention program, which addresses aspects as diverse as: knowledge about GBV and legislation; the resources available to adolescents in the event of witnessing or suffering violent relationships; and the enhancing of awareness regarding abusive and violent relationships, myths of romantic love, gender stereotypes, sexism and everyday micro-sexism. The program seeks to intervene in successive years on attitudes of acceptance or justification for violence in relationships and on the development of the social and emotional skills needed to ensure a dating relationship free from violence. The combination of these aspects in the intervention (attitudes, beliefs, knowledge, and personal skills) is considered essential to secure true prevention of GBV and DV (Foshee et al., 1996). Another aspect to be considered is the strong involvement and commitment of the master and psychology degree students in conducting the intervention, as well as the instructor training process corresponding to each session. The enthusiasm, involvement and preparation of the interveners is one of the key factors ensuring the efficacy of preventive programs (Dane and Schneider, 1998). Another strong point is the assessment of a possible differential gender effect of the program, which was not observed in our case.

## Conclusion

The results of the program in its first year of intervention have been positive and encourage continued implementation of the program and assessment of its effects over the next 2 years of intervention – with necessary confirmation of its long-term effects.

## Data Availability Statement

The raw data supporting the conclusions of this article will be made available by the authors, without undue reservation.

## Ethics Statement

The studies involving human participants were reviewed and approved by the Clinical Research Ethics Committee from the King Juan Carlos University (code: 0404201806318). Written informed consent to participate in this study was provided by the participants’ legal guardian/next of kin.

## Author Contributions

LV, HT-C, and YP-R conducted the evaluation of methodology, supervisions, drafting the manuscript, and revisions for important intellectual content. YP-R performed data analysis. AA-R performed data collection, administrative, technical, and material support. All authors have read and approved the final manuscript, generated the initial research questions, developed the research protocol, and defined the inclusion and exclusion criteria.

## Conflict of Interest

The authors declare that the research was conducted in the absence of any commercial or financial relationships that could be construed as a potential conflict of interest.

## Publisher’s Note

All claims expressed in this article are solely those of the authors and do not necessarily represent those of their affiliated organizations, or those of the publisher, the editors and the reviewers. Any product that may be evaluated in this article, or claim that may be made by its manufacturer, is not guaranteed or endorsed by the publisher.

## Abbreviations

GV: gender violence
GBV: gender-based violence
VAW: violence against women
DV: dating violence.
